# Supplementary figures and images for: Hierarchical regulation of autophagy during adipocyte differentiation
Source: PLoS One. 2022 Jan 26;17(1):e0250865. doi: 10.1371/journal.pone.0250865 (PMC8791469; doi:10.1371/journal.pone.0250865)

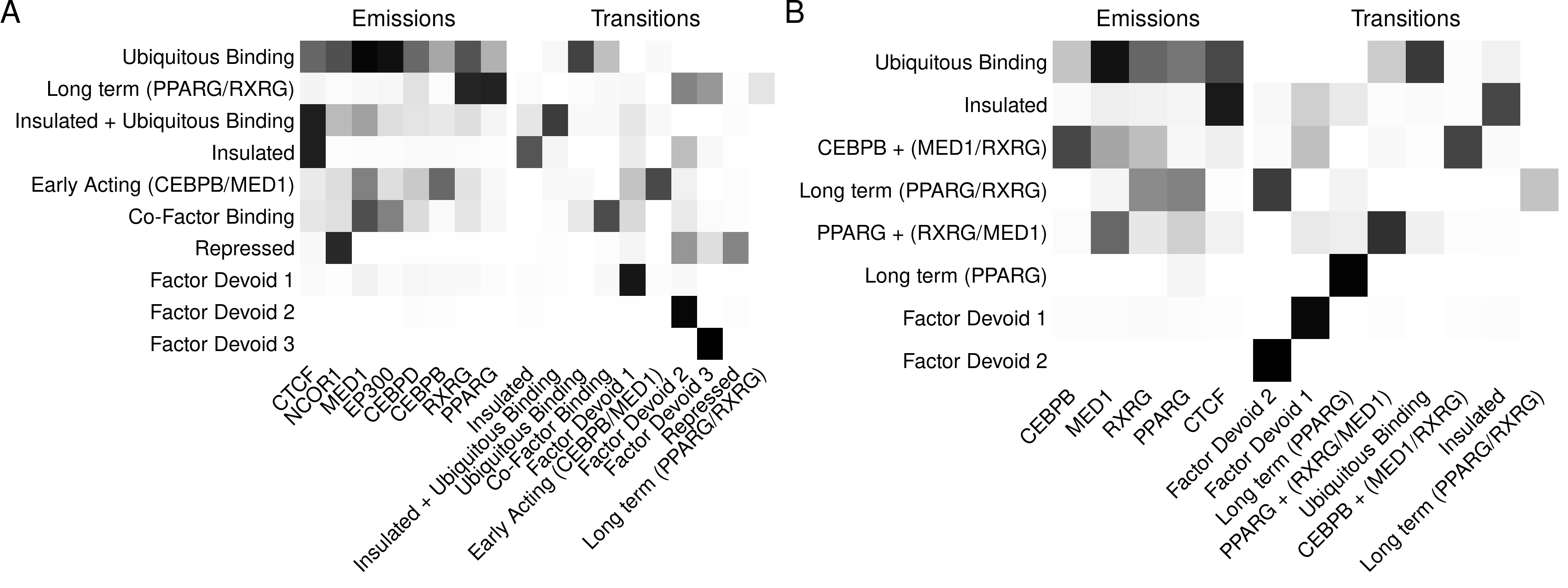

Supplement: S1 Fig — We curated a dataset of publicly available transcription factors, co-factors, and DNA-binding proteins ChIP-seq samples in MDI-induced 3T3-L1 at different time points (Table 2). Binarized binding signals were used to indicate the presence or absence of regulators at 100 bp windows of the chromatin. A multi-variate model of combinations of regulators was built to summarize A) ten states in the early (up to 48 hr) stage or B) eight states in the full course (up to 10 days) of differentiation. Emission, the probability of each marker being at a given state. Transition, the transitional probability of a given state from/to another. white, low, and black, high probability. (PNG) [file pone.0250865.s001.png]

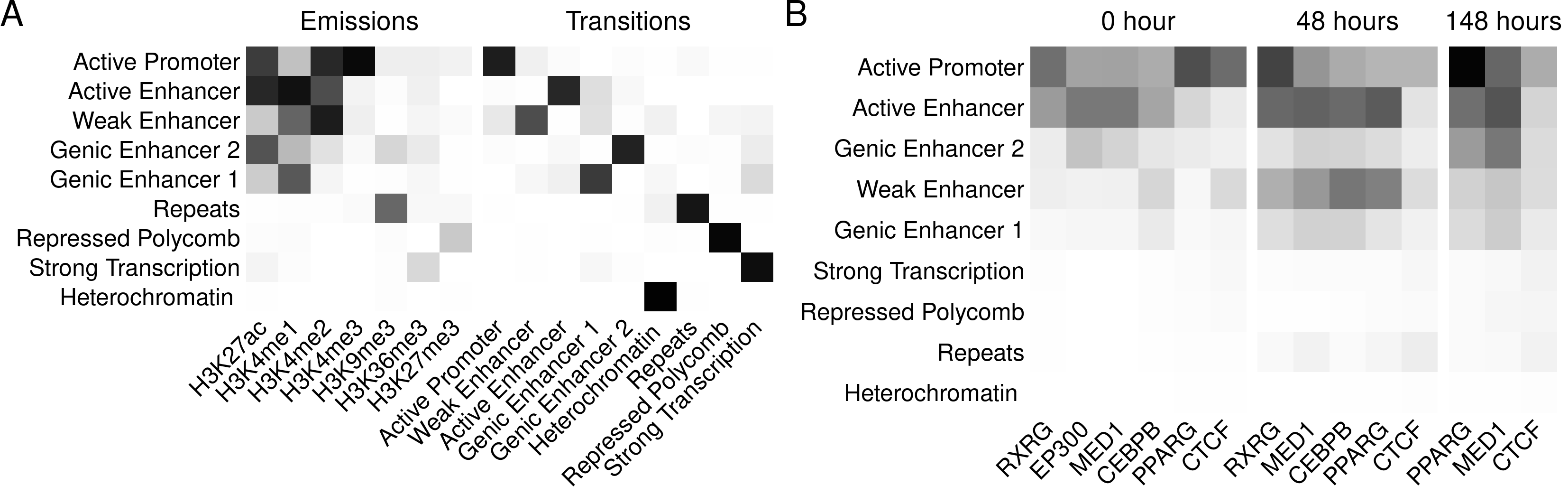

Supplement: S2 Fig — We curated a dataset of publicly available histone modification ChIP-seq samples in MDI-induced 3T3-L1 at different time points (Table 3). Binarized binding signals were used to indicate the presence or absence of histone markers at 200 bp windows of the chromatin. A multi-variate model of combinations of markers was built to summarize nine states during the course of differentiation. A) Emission, the probability of each marker being at a given state. Transition, the transitional probability of a given state from/to another. white, low, and black, high probability. B) Binding peaks of adipogenic regulators were used to identify gene targets at different time points of adipocyte differentiation from publicly available ChIP-seq sample. The fractions of overlap between the regulators’ binding sites and the chromatin states are shown as color values (white, low, and black, high). (PNG) [file pone.0250865.s002.png]
