# Supplementary material for: Hierarchical regulation of autophagy during adipocyte differentiation
Source: PLoS One. 2022 Jan 26;17(1):e0250865. doi: 10.1371/journal.pone.0250865 (PMC8791469; doi:10.1371/journal.pone.0250865)
Supplement: Attachment — Submitted filename: Responsestoreviewers_1.docx [file pone.0250865.s003.docx]

## Reviewer #1

### Evidence, reproducibility and clarity

This study builds on prior work by the same group, focusing on transcriptional regulation of autophagy genes during 3T3-L1 adipogenesis. The current paper utilizes older datasets. In general, it is not really clear what this paper adds to what we learned from the previous paper.

In a previous study, we showed that two important adipogenic transcription factors (PPARG and CEBPB) regulate the expression of autophagy-related genes either directly or indirectly through other specific transcription factors. In this study, we investigated the spatial and temporal arrangement of those two factors and their cofactors. We believe that these findings elaborate the mechanisms of the transcription regulation of autophagy by these transcription factors and co-factors. In addition, we showed that these regulatory links are potentially involved in the induction of specific forms of autophagy, have a strong temporal dimension, and work through specific effectors (other factors or kinases) compared to the regulation of lipogenesis. In response to the reviewers' comments, we revised the Introduction & Discussion section to stress the value added by the study to previous work.

The major issues are as follows:

1. There is no attempt to measure autophagy (or mitophagy, or aggrephagy, etc...) at any time point to correlate an actual functional outcome with the changes in gene expression. This severely limits the authors' ability to say anything about whether the gene expression changes studied have any relationship to actual autophagy.

Our analysis is based on two data types (RNA and ChIP-seq). We reported observations on the changes in gene expression, DNA-binding, and the correlation of the two. We don't make any explicit claims on the function, since our analysis doesn't include any functional data. We recognize that this kind of observation is only valid insofar as phenotypes reflect the underlying gene expression. We used the gene set enrichment and over-representation analysis to statistically quantify these observations at the gene set level, with is more likely to correspond to biological functions than claims based on changes in the expression of individual genes. We explained this view with the other limitations of the study in the Discussion section.

2. The gene sets that are used to define autophagy and the other phenomena (e.g., mitophagy) are never defined, unless I just missed it.

We use the gene ontology terms (as defined in the [org.Mm.db.eg](http://org.Mm.db.eg) and GO.db packages) to define the gene sets of interest. We revised the relevant section of the Methods section to clarify the selection process and define the ontology terms.

3. In general, PPARg and C/EBPs bind to cis-regulatory regions of almost every gene that is induced during adipogenesis. This is not unique to autophagy genes (as shown by the nearly identical patterns seen for lipogenic genes), so it's hard to understand why the authors focus on autophagy genes.

Our initial goal was to study the regulation of autophagy during adipogenesis mainly through gene expression data. In previous work, we found that autophagy genes are regulated as part of the transcriptional program and adipogenic transcription factors and co-factors were the obvious candidates as drives of the gene expression. As a result, we used ChIP-seq data to identify binding patterns and correlate them with changes in gene expression. The role of these factors is well described in the case of the processes that are directly related to adipogenesis and lipogenesis. Therefore, we believe it is useful to study these patterns of regulation in the case of autophagy. We also found key differences between the way autophagy and lipogenesis genes are regulated by these factors. Namely, the autophagic response to the adipogenic induction is biphasic and the adipogenic factors target other factors and kinases more specifically. We clarified study motivation and focus on autophagy genes in the Introduction section.

4. The authors suggest that co-factors bind to key loci before transcription factors. I don't follow the argument. First, some of the co-factors listed (such as RXR and C/EBPb), are in fact transcription factors, so the distinction doesn't hold. Second, the co-factors that do not bind DNA directly, like MED1, need a TF to localize to DNA by definition. If MED1 is not working through PPARg or C/EBPs at early time points, how is it binding DNA?

Indeed, CEBPB is a transcription factor and is recognized as such throughout the manuscript. Although RXRG was also reported to function as a transcription factor, in many cases it was also reported to work in partnership with PPARG. We don't hold strong views on the distinction between the factors and co-factors. For the purposes of this study, we treat either by their previously reported functional role. In the sentence referred to we didn't mean CEBPB is a co-factor, but rather the described trend applies to the two factors and to CEBPB as a transcription factor. We corrected the sentence for clarity. Since the study includes only a handful of regulators, several others must be at work which probably plays a role in the DNA-binding. ChIP-seq data can show that a particular protein is associated with a piece of DNA but doesn't exclude other proteins on the exact site or specific the form of direct or indirect binding. Our observation was, on many autophagy gene regions, co-factors appeared to associate the DNA earlier in time than the transcription factor. Those were not necessarily the same binding regions, but regions assigned to the same gene. We discussed the distinction of the factor/co-factor issue in the manuscript and explained how either was used in our study.

### Significance

I am an adipose biologist, interested in gene expression in fat cells during differentiation. The key conclusions are already available in the authors' prior work.

In a previous study, we showed that two important adipogenic transcription factors (PPARG and CEBPB) regulate the expression of autophagy-related genes either directly or indirectly through other specific transcription factors. In this study, we investigated the spatial and temporal arrangement of those two factors and their cofactors. We believe that these findings elaborate the mechanisms of the transcription regulation of autophagy by these transcription factors and co-factors. In addition, we showed that these regulatory links are potentially involved in the induction of specific forms of autophagy, have a strong temporal dimension, and work through specific effectors (other factors or kinases) compared to the regulation of lipogenesis. In response to the reviewers' comments, we revised the Introduction & Discussion section to stress the value added by the study to previous work.

## Reviewer #2

### Evidence, reproducibility and clarity

Overall, the conclusions of the manuscript are not well supported by key data. Particularly, the text and figures are not clear and accurate. For instance, there are numerous grammatical errors throughout the manuscript and some of claims in the manuscript are very speculative and not supported by the data present in the manuscript. Also, most figures and figure legends are not accompanied by sufficient information required to understand what each figure stands for. Given that the authors analyze different types of high-throughput sequencing data, it is needed to provide sufficient information on the criteria used for analyzing correlation between two different types of dataset (e.g., RNA-seq vs. TF ChIP-seq) in the method section. However, this information is absent in the method section of the current manuscript. Therefore, it is suggested that the manuscript needs to be extensively amended to support the current conclusions.

We made the following changes in response to the reviewer's comments

- Revised the manuscript for clarity and language
- Corrected the figures
- Expanded the figure legends to include details of they were generated
- Addressed the issue of using two types of data in the Methods and the Discussion section

Several major issues with this manuscript are describe below.

1. The main hypothesis of the manuscript is that a hierarchical arrangement of adipogenic TFs and related co-factors would regulate autophagy-related gene expression during adipogenesis. In particular, all of data presented in the current manuscript are focused on the correlation of PPARgamma and CEBPbeta bindings with differential expression of autophagy related genes during adipogenesis although, in addition to PPARgamma and CEBPbeta there are multiple adipogenic transcription factors (e.g., CEBPalpha, CREB, and KLF2 etc.) that play key roles in adipogenesis. To support the authors' claims that these two particular TFs are important for the expression of autophagy related genes, it should be shown whether PPARgamma and CEBPbeta bindings are more closely associated with differential expression of autophagy related genes compared to other adipogenic TFs during adipogenesis.

Our analysis doesn't rule out the involvement of other transcription factors in regulating autophagy genes. We observed significant binding and gene expression changes during adipogenesis that seem to be correlated with those two factors. In addition, the two factors and their co-factors explain a significant portion of the variance over time. We discussed this issue in the limitations of the study in the Discussion section.

2. In Figure 1B, the statement regarding to "lipid metabolic process" in the result section is ambiguous. Does "lipid metabolic process" mean "lipogenesis" presented in Figure 1B? If so, because the enrichment score of lipogenesis falls down to around -1 at 24 hr in Figure 1B, the statement of "always represented in the up-regulated ranks in the list of genes" does not appear to be proper interpretation of Figure 1B. If "lipid metabolic process" does not mean "lipogenesis", the description appears to be very irrelevant to Figure 1B that shows the changes in enrichment score of "lipogenesis" but not "lipid metabolic process" during adipogenesis. Therefore, to support the authors' claims related to Figure 1B, it is strongly recommended to either revise the statement or change Figure 1B to include data related to "lipid metabolic process".

We revised the sentence and the figure to be consistent. The curve shown in the figure is for the "lipid metabolic process" gene ontology term. It's true that after 24 hours the enrichment score of the lipid metabolic process term is negative. We believe however that the trend is clear and otherwise. Although we cannot explain the reason this anomaly with certainty, it's to be noted that the scores at 24 hours are negative but the corresponding p-values are large (> 0.05).

3. In Figure 1C, what is the control group used to identify up- or down-regulated genes? Is negative siRNA group used as control to identify DEGs? Or is the expression of preadipocyte used to determine DEGs? Because genetic manipulation can impact on overall transcriptome, it is appropriate to use negative siRNA group as control group. Therefore, if the expression of preadipocyte is used as control in Figure 1C, it should include the data of DEGs that was identified using negative siRNA group as a control.

The figure shows the differential expression between knockdown and negative controls (siRNA/shRNA). We stated the design in detail in the Methods and the figure legends.

4. The labels of x- and y-axis in most figures appear to be inaccurate and speculative. For instance, the y labels of Figure 1A and 1C, do not match with the data description in Result section. As the number in the y-axis represents the number of autophagy related genes that are either down- or up-regulated during adipogenesis, the label should be changed to "the number of genes" rather than "gene expression". Also, in Figure 2A and 2B, there is no information related to what x- and y-axis stand for. Does the number in the y-axis indicate the time where PPARgamma and CEBPbea bindings are analyzed? If so, what the x-axis stands for?

We corrected the figures to better reflect the data they represent. In Figure 1A & C, the numbers shown of the y axis are those of the differentially expressed genes as pointed out by the reviewer. We also revised the labels in Figure 2. The figure shows the average expression at different time points (x-axis) of the transcription factor targets (as identified at different time points, y-axis)

5. It is hard to understand why the number in the y-axis of Figure 2A and 2B and x-axis of Figure 6A is not arranged in chronological order. The order of the number in y-axis of Figure 2A is 0, 24, 168, 144, 48, 96, 72 (hr), which appears to be very inappropriate. Similarly, the order of the number in y-axis of Figure 2B is 6, 4, 0, 2, 48 (hr). As y and x-axis in other figures are arranged in chronological order, it is strongly recommended to reorganize the data related to Figure 2A and 2B to fit y-axis that is organized in chronological order. More importantly, the authors should reconsider and carefully revise their claims suggesting the prolonged effect of PPARgamma and transient effect of CEBPbeta on autophagy-related genes during adipogenesis which is drawn from current form of Figure 2A, 2B and Figure 6A.

In figure 2, the heatmap is arranged by hierarchical clustering based on the euclidean distances. We believe this clustering highlights the observations we made in the interpretation in the results section, as it groups similar expression profiles together. In Figure 6A, the bars are ordered by descendingly from the largest to the smallest set size. We also believe this highlights the key finding in the figure. In both cases, we explained the reasoning of the ordering in the figure legends.

### Significance

In the current manuscript, the authors demonstrated the significance of a hierarchical arrangement between adipogenic transcription factors (TFs) and co-factors in the regulation of autophagy-related gene expression by integrating multiple layers of high-throughput sequencing data including RNA-seq, histone ChIP-seq and TF ChIP-seq data.
